# Role of Mesenchymal Stem Cells in Counteracting Oxidative Stress—Related Neurodegeneration

**DOI:** 10.3390/ijms21093299

**Published:** 2020-05-07

**Authors:** Cristina Angeloni, Martina Gatti, Cecilia Prata, Silvana Hrelia, Tullia Maraldi

**Affiliations:** 1 School of Pharmacy, University of Camerino, Via Gentile III da Varano, 62032 Camerino, Italy; cristina.angeloni@unicam.it; 2 Department of Surgery, Medicine, Dentistry and Morphological Sciences, University of Modena and Reggio Emilia, Via del Pozzo 71, 41124 Modena, Italy; martina.gatti@unimore.it (M.G.); tullia.maraldi@unimore.it (T.M.); 3 Department of Pharmacy and Biotechnology, Alma Mater Studiorum—University of Bologna, Via Irnerio 48, 40126 Bologna, Italy; 4 Department for Life Quality Studies, Alma Mater Studiorum—University of Bologna, Corso d’Augusto 237, 47921 Rimini, Italy; silvana.hrelia@unibo.it

**Keywords:** mesenchymal stem cells, oxidative stress, neurodegeneration, secretome

## Abstract

Neurodegenerative diseases include a variety of pathologies such as Alzheimer’s disease, Parkinson’s disease, Huntington’s disease, amyotrophic lateral sclerosis, and so forth, which share many common characteristics such as oxidative stress, glycation, abnormal protein deposition, inflammation, and progressive neuronal loss. The last century has witnessed significant research to identify mechanisms and risk factors contributing to the complex etiopathogenesis of neurodegenerative diseases, such as genetic, vascular/metabolic, and lifestyle-related factors, which often co-occur and interact with each other. Apart from several environmental or genetic factors, in recent years, much evidence hints that impairment in redox homeostasis is a common mechanism in different neurological diseases. However, from a pharmacological perspective, oxidative stress is a difficult target, and antioxidants, the only strategy used so far, have been ineffective or even provoked side effects. In this review, we report an analysis of the recent literature on the role of oxidative stress in Alzheimer’s and Parkinson’s diseases as well as in amyotrophic lateral sclerosis, retinal ganglion cells, and ataxia. Moreover, the contribution of stem cells has been widely explored, looking at their potential in neuronal differentiation and reporting findings on their application in fighting oxidative stress in different neurodegenerative diseases. In particular, the exposure to mesenchymal stem cells or their secretome can be considered as a promising therapeutic strategy to enhance antioxidant capacity and neurotrophin expression while inhibiting pro-inflammatory cytokine secretion, which are common aspects of neurodegenerative pathologies. Further studies are needed to identify a tailored approach for each neurodegenerative disease in order to design more effective stem cell therapeutic strategies to prevent a broad range of neurodegenerative disorders.

## 1. Oxidative Stress and Neurodegenerative Diseases

Neurodegenerative diseases are a heterogeneous class of disorders characterized by a slow and chronic loss of different neuronal populations in the central nervous system (CNS). Alzheimer’s disease (AD), Parkinson’s disease (PD), Huntington’s disease (HD), amyotrophic lateral sclerosis (ALS), and spinocerebellar ataxia (SCA) are the most common neurodegenerative diseases [1]. They are a primary public health problem, affecting tens of millions of people worldwide [2]. Although their complex and multifactorial etiology has not been fully elucidated, increasing evidence suggests a causative role of oxidative stress in the onset and development of these disorders [3,4,5,6]. 

**Figure 1:**
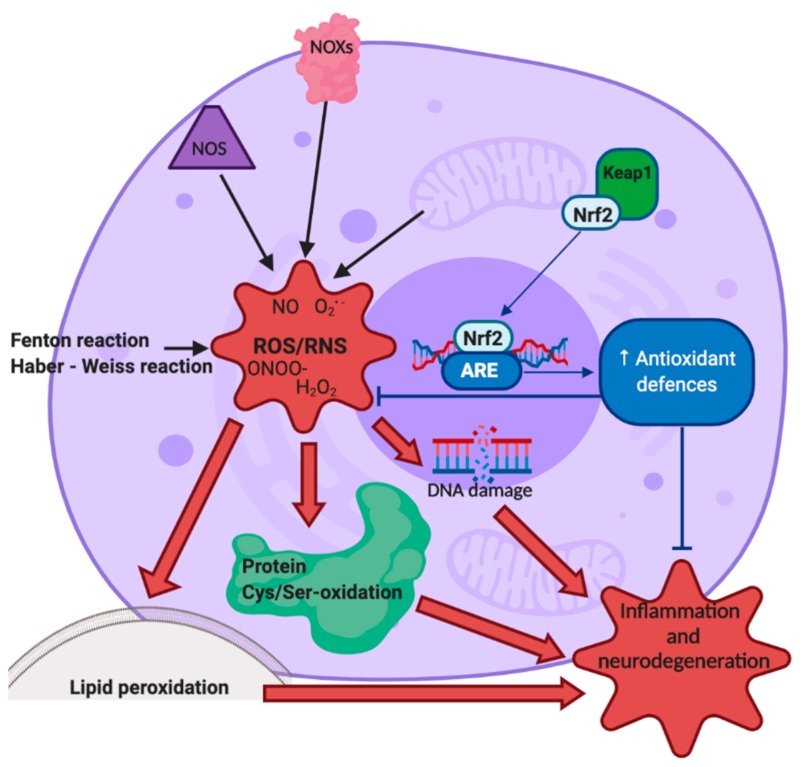
Schematic representation of oxidative stress sources and effects. Image created with BioRender.com.

Oxidative stress, produced by an imbalance between reactive oxygen species (ROS) and the endogenous antioxidant defense system [7], plays a key role in neurodegeneration as the brain is characterized by an elevated oxygen consumption, high levels of iron and copper, involved in ROS production, elevated content of polyunsaturated fatty acids, and low antioxidant defenses [1,8]. A schematic representation of causes and consequences of oxidative stress is shown in Figure 1. ROS can be produced by the Fenton and Haber–Weiss reactions, in which redox-active metals interact with oxygen species, or by the reactions catalyzed by NADPH oxidases or nitric oxide synthase (NOS). The most investigated ROS are hydrogen peroxide (H_2_O_2_), superoxide (O_2_^•−^), and hydroxyl radical (•OH). ROS are principally generated in the mitochondria, as they are the largest intracellular oxygen consumer during electron transfer in mitochondrial respiratory chain by the interaction between the leaking electrons and molecular oxygen [9]. Oxidative stress can also be induced by reactive nitrogen species (RNS) [10]. RNS are produced by the fast reaction of O_2_^•−^ with nitric oxide (NO), resulting in the generation of peroxynitrite (ONOO^−^) that can trigger DNA fragmentation and lipid peroxidation [11,12]. Three different isoforms of NOSs, namely endothelial (eNOS), neuronal (nNOS), and inducible (iNOS), generate NO. It has also been observed that NO inhibits complexes I and IV of the mitochondrial electron transport chain, inducing ROS generation [4]. 

Cells respond to oxidative stress by inducing antioxidant enzymes that can neutralize ROS counteracting cell damage [13,14]. In particular, nuclear factor erythroid 2-related factor 2 (Nrf2) enhances the expression of these antioxidant enzymes thanks to its binding to a specific sequence in the promoter region of these genes called antioxidant response element (*ARE*) [15,16]. In normal physiological condition, Nrf2 is sequestered in the cytoplasm by Kelch-Like ECH-Associated Protein 1 (Keap1) that promotes ubiquitination and degradation of Nrf2 in the proteasome. Nevertheless, when ROS or electrophiles modify the nucleophilic cysteine sulfhydryl groups on Keap1, Nrf2 is released and it moves to the nucleus [17]. Furthermore, Nrf2 also exerts anti-inflammatory activity and modulates both biogenesis and mitochondrial function [18,19,20]. On these bases, Nrf2 is considered an interesting therapeutic target in counteracting neurodegeneration [21,22].

In the following paragraphs, we describe the impact of oxidative stress in different neurodegenerative diseases.

### 1.1. Alzheimer’s Disease 

Alzheimer’s disease (AD) is the main cause of dementia and affects about 5.7 million Americans and over 30 million people worldwide [23]. It is characterized by the degeneration and irreversible loss of neurons and synapses in the brain impairing memory, personality, and cognitive functions. Despite the several hypotheses explored to explain the origin of AD, the most accepted ones are the extracellular deposition of Aβ protein, a 40–42-amino acid peptide generated by proteolytic cleavages of the amyloid-protein precursor (APP), and intraneuronal neurofibrillary tangles (NFTs) of hyperphosphorylated tau protein [24,25]. Another recognized hallmark of AD is oxidative stress that, together with low levels of antioxidants and antioxidant enzymes, has been reported in the brains of AD patients [26,27,28,29]. Oxidative stress, Aβ proteins, and NFTs are involved in a complicated relationship: it has been suggested that oxidative stress induces Aβ aggregation and facilitates the phosphorylation and polymerization of tau, and, in turn, protein deposition increases oxidative stress, creating a vicious cycle [29]. 

Different lines of evidence suggest that Aβ induces oxidative stress. In particular, Aβ1–42 inserts as oligomers into the bilayer and serves as a source of ROS inducing oxidative stress and initiating lipid peroxidation [30]. Aβ is mainly accumulated in the extracellular regions but in lower amount is also found in neurons [31]. Aβ in mitochondria may impair mitochondrial respiration, increasing ROS generation, reducing ATP synthesis, and contributing to an altered calcium homeostasis [28,29]. High level of mitochondrial ROS triggers mitochondrial DNA mutations that, in fact, are increased in AD patients [29]. Moreover, Aβ amyloid is able to directly initiate ROS production by the activation of NADPH oxidase [32]. Consequently, ROS overproduction triggered by Aβ proteins can alter cellular signaling pathways causing tau hyperphosphorylation via the activation of p38 mitogen activated protein kinases [1]. 

It has been evidenced a strict relation between tau pathology and oxidative stress. Oxidative stress may promote hyperphosphorylation and polymerization of tau through the oxidation of fatty acids that are elevated in AD and have been shown to increase tau polymerization [33]. It has been observed that tau phosphorylation can be increased by p38 MAPK, that, in turn, is activated by oxidative stress [34]. On the other hand, the overexpression of tau protein increases cell vulnerability to oxidative stress, and this has been related to the depletion of peroxisomes [35]. 

Oxidative stress can also be promoted by protein glycation in AD [36]. Glycation is an endogenous process, triggered by high levels of glucose or by reactive endogenous aldehydes such as methylglyoxal or glyoxal, leading to the generation of a group of species known as advanced glycation end products (AGEs). Remarkably, high AGE levels have been detected in the brains of AD patients and glycosylation increased Aβ and NFTs proteolytic resistance [36,37]. 

Another critical ROS source in AD brain is activated microglia. Within the CNS, microglia are considered as the resident macrophages of the brain [38]. They rapidly respond to neuroinflammation and damage interacting with astrocytes and neurons, assuming phagocytic phenotypes and releasing pro-inflammatory cytokines, nitric oxide (NO), and ROS [39,40]. Microglia participate in Aβ clearance [41], and, from this point of view, they can be both beneficial and deleterious: they counteract Aβ deposition, but high levels of Aβ trigger a prolonged inflammatory condition that leads to an overproduction of inflammatory mediators. Moreover, neuroinflammation causes an increase in NO synthesis by microglia and astrocytes. High level of NO, in the presence of ROS, generates ONOO¯ and other reactive nitrogen species that are crucial contributors to oxidative stress in AD [42].

Different studies have suggested a relation between oxidative stress and glutamate excitotoxicity in the neurodegenerative process of AD [43]. Glutamate is a neurotransmitter that has a critical role in learning and in the formation of memory through long-term potentiation (LTP) [44]. However, a condition called glutamate excitotoxicity can occur when extracellular glutamate reaches high concentrations leading to cell death caused by the excessive activation of the n-methyl-d-aspartate receptor [45]. The impairment of these signaling pathways can trigger synaptic dysfunction, apoptosis, and activation of the calcium-dependent pathway of calpain [46,47]. Mitochondrial dysfunction has been indicated as one of the primary events in glutamate excitotoxicity [48]. Oxidative stress triggered by mitochondrial dysfunction can damage neurons promoting the release of glutamate. Elevated levels of glutamate in the synaptic cleft activate the receptors on adjacent neurons, and this causes an excessive and sustained rise in free Ca^2+^ within them and therefore promoting a vicious circle of neuronal damage [49].

**Figure 2:**
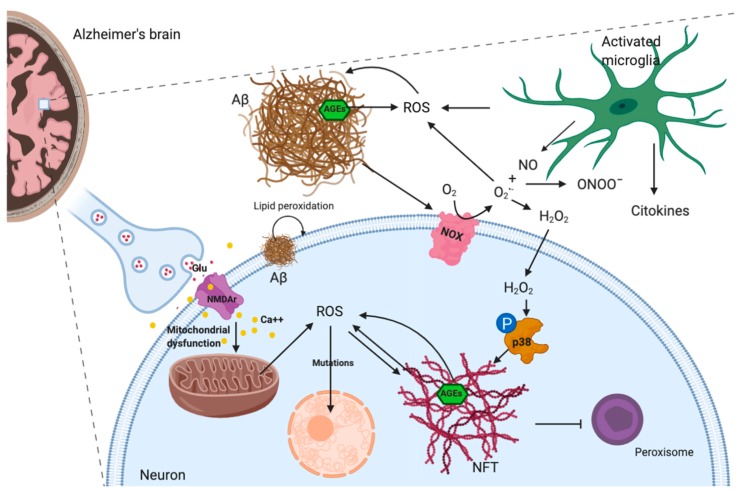
Schematic representation of ROS sources and their molecular targets in AD. Image created with BioRender.com.

In conclusion, oxidative stress has a fundamental role in AD and affects different molecular pathways involved in AD brain cells (Figure 2). At the moment, what should be better understood is whether oxidative stress plays a causative role in the onset of AD or is only a byproduct of other AD mechanisms.

### 1.2. Parkinson’s Disease

PD is the most common neurodegenerative disease after AD and affects 1–2% of the population over 65 and this rate increases to 4% in individuals above 85 years of age [50]. It is characterized by dopaminergic neuron loss in the substantia nigra pars compacta [51] and decreased dopamine (DA) levels in the nigrostriatal DA pathway in the brain [52]. The decrease in DA is associated with several sensory and motor impairments, in particular tremor, rigidity, and akinesia [53]. One distinctive characteristic of PD is the abnormal accumulation of Lewy bodies and Lewy neurites within neurons [54]. Insoluble α-synuclein fibrils are the principal constituent of both Lewy bodies and Lewy neurites [55].

Increasing evidence suggests that oxidative stress plays a major role in PD [4,56]. Even if the exact molecular mechanism causing dopaminergic cell death remains elusive, many studies observed that the substantia nigra pars compacta of PD patients is characterized by highly oxidized lipids, proteins, and DNA, together with low levels of glutathione [42]., Notably, in contrast to other regions of the brain, the substantia nigra is highly subjected to ROS attack because neurons are exposed to additional oxidative stress due to their dopamine metabolism, low content of antioxidants, and high iron concentration [57]. Dopamine is oxidized by monoamine oxidases A and B with the production of hydrogen peroxide. In addition, dopamine is subjected to auto-oxidation with the production of quinones, peroxide, and oxy-radicals [58]. In healthy and young individuals, the DA transporter (DAT) protects dopaminergic neurons against oxidized DA [57]. Oxidized DA is taken up by DAT into the nerve terminal, where vesicular monoamine transporter 2 (VMAT2) repackage DA in the synaptic vesicles. Unfortunately, a significant age-related decline in the DAT has been observed, typically around 5.6–6.1% per decade, which has been associated to dopaminergic neuron loss in the substantia nigra and increase of oxidative stress [59]. DA oxidation to o-quinones is also an essential event required for neuromelanin synthesis [60]. In healthy individuals, neuromelanin acts as a protective factor in the substantia nigra; however, in PD patients, the modification of the composition and density of neuromelanin is suggested to increase ROS production and iron concentration [61,62]. It has been observed that α-synuclein accumulates on neuromelanin, leading to a significant reduction in neuromelanin density within substantia nigra of dopaminergic neurons [4], altering the neuroprotective function of neuromelanin in PD. In fact, neuromelanin has a fundamental role in blocking reactive iron, forming stable complex with it, then preventing iron toxicity [60]. Different studies have demonstrated higher levels of iron in the brain of PD patients [63,64], which has also been associated to the impaired iron transport into the mitochondria in dopaminergic neurons in PD patients [65,66]. 

Another important oxidative stress production site in PD is the mitochondria as suggested by the decrease of Complex I activity in the respiratory chain in the substantia nigra in PD [67]. As underlined above, together with an increase of oxidative stress in postmortem PD substantia nigra, a reduction in the levels of GSH has been observed [68,69,70]. GSH is a fundamental endogenous antioxidant and a decrease in its concentration inevitably leads to an enhanced ROS production. 

Moreover, extensive inflammation has been reported in PD, as demonstrated by high levels of pro-inflammatory cytokines such as IL-1b, TNF-a, IL-2, and IL-6, measured in PD patients, in the post-mortem brain, and in serum and cerebrospinal fluid in vivo [71]. Since inflammation is associated to a huge release of ROS [72], and as PD patients are exposed to a long-term strong chronic inflammation in the brain, oxidative stress can be exacerbated in these patients [72]. 

**Figure 3:**
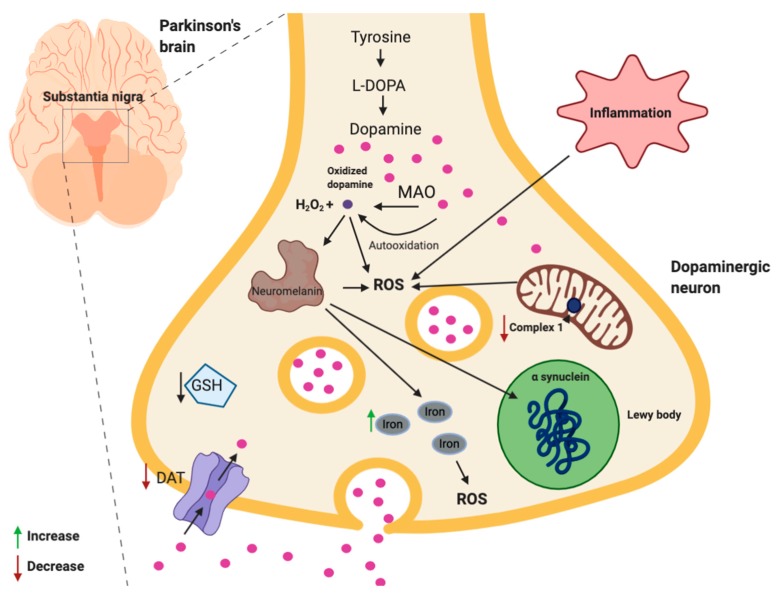
Schematic representation of ROS sources and their molecular targets in PD. Image created with BioRender.com.

Summarizing, similar to AD, oxidative stress seems to have both a causal role and being a consequence of different pathological conditions in PD (Figure 3). In this context, the auto-oxidation of dopamine is an additional element that contribute to oxidative stress.

### 1.3. Amyotrophic Lateral Sclerosis

Among the various neurodegenerative diseases, ALS is the most common neuromuscular disorder that leads to lethal respiratory paralysis within 3–5 years of diagnosis [73]. Usually it occurs after 50 years of age, has an incidence of around five people per 100,000 worldwide, and is more frequent in men [74]. ALS can be classified as either sporadic or familial form, with the majority of cases among the population being sporadic. The term sporadic refers to ALS that has no clear genetic relationship and occurs without a family history of ALS. On the other hand, 10% of cases can be familial and triggered by mutations in some genes, such as *SOD1, TDP43, C9orf72*, and *FUS* [73]. 

The dominant pathological characteristic of ALS is the occurrence of inclusions in the cytoplasm or aggregates into motor neurons and nearby oligodendrocytes. The principal aggregates present in patients suffering ALS are ubiquitinated aggregates and can be either Lewy body-like hyaline inclusions or skein-like inclusions [75]. Ubiquitinated aggregates observed in ALS can induce ROS generation both in the cytosol and in mitochondria [76,77,78]. 

In turn, oxidative stress might alter protein structure, producing abnormal protein inclusions, generating in this way a detrimental loop [79].

Different studies showed the involvement of several factors in ALS, such as neuroinflammation, mitochondrial dysfunction, excitotoxicity, stress of the endoplasmic reticulum, and oxidative stress [80]. Increased levels of protein oxidation, nitration, and carbonylation, together with lipid peroxidation, have been widely observed in familial and sporadic ALS patients and in different models of the disease [81,82,83], indicating a crucial role of oxidative stress in the pathogenesis of ALS [84]. 

The impairment of the activity of mSOD1 and other ALS-linked proteins, such as mutant TDP-43, increases ROS and triggers oxidative stress [85,86]. 

Excitotoxicity and oxidative stress are strictly related in ALS [87]. As previously underlined, neuronal excitotoxicity is characterized by an elevation of cytosolic free calcium that, in turn, activates calcium-dependent enzymes, such as proteases and enzymes including xanthine oxidase, phospholipase A2, and NOS that can produce ROS and RNS [88]. Moreover, motor neurons are especially sensitive to increases in cytosolic free calcium levels because, compared to other kinds of neurons, they are rather poor in some proteins that bind calcium such as calbindin D-28k and parvalbumin [89]. 

Neurons persist throughout the existence of an organism and, for this reason, the preservation of healthy mitochondria is crucial for the survival and function of neurons. It is thus not surprising that mitochondrial dysfunction has been associated not only to AD and PD but also to ALS [90]. Indeed, damaged mitochondria are an early change observed in motor neurons of ALS patients [91,92]. This damage can be due to different factors including the interaction of proteins linked to familial and sporadic ALS with mitochondria [93,94,95]. ALS associated mitochondrial dysfunction unavoidably leads to the production of ROS and to oxidative stress.

In ALS, another cause of ROS production is inflammation, observed in both patients suffering ALS and mSOD1 mice [87]. Indeed, a strong increase in pro-inflammatory markers such as interleukin-6 (IL-6), monocyte chemoattractant protein-1 (MCP-1), IL-8, and cyclooxygenase-2 (Cox-2) is present in ALS [96,97,98,99,100]. It has also been evidenced that macrophages infiltrate ventral spinal roots, peripheral motor nerves and skeletal muscles in ALS mouse models [101,102]. Therefore, activated macrophages might also contribute to ROS production via NADPH oxidases in axons and muscle in ALS [87]. Moreover, microgliosis is an important contributor to neurodegeneration as well as oxidative stress. Indeed, in human spinal cord samples of ALS mouse model, high NOX2 expression was detected in microglia [103]. The authors demonstrated that NOX inhibition with thioridazine significantly decreased superoxide levels in the spinal cord and dampened the increase of microglia markers in SOD1(G93A) mice, thus resulting in an immediate and temporary enhancement of motor performance. Furthermore, diminishing ROS produced by microglia by treatment with another NOX inhibitor apocynin or by elimination of NOX has been reported to extend survival in ALS mice, reviving the proposal that ROS mediate ALS pathogenesis [104]. 

In conclusion, oxidative stress plays a fundamental role in ALS being both cause and consequence of other impaired functions. ALS, indeed, is characterized by a complex network of molecular mechanisms underpinning neurodegenerative processes, in which disturbances in redox homeostasis, as common mechanism behind oxidative stress, inflammation, and excitotoxicity, play a fundamental role.

### 1.4. Oxidative Damage of Retinal Ganglion Cells

Retinal ganglion cells (RGCs) are neurons located near the inner retina surface, that receive visual information from photoreceptors, allowing the connection between brain and visual world. There are over a million RGCs in the eyes, classified according to different physiological, morphological, and molecular criteria [105]. RGCs represent one of the central cell types damaged in many ocular neurodegenerative diseases, characterized by oxidative stress, such as diabetic retinopathy [106,107], glaucoma [108,109], and retinal ischemia-reperfusion injury [110,111]. It has been proved that oxidative stress plays a pivotal role in the loss of RGCs in several eye diseases. Moreover, aging process aggravates the injury in RGCs through several processes, such as DNA damage, oxidation of proteins, apoptosis, cell death, etc. As previously reported, the Nrf2/Keap1/ARE signaling pathway is one of the key protection mechanisms against oxidative stress and the inducers of this signaling pathway can be considered as emerging target with a neuroprotective effect in RGCs [112,113,114]. Indeed, a recent review discusses the effect of several Nrf2 inducers that protect RGCs from oxidative stress-induced optic neuropathies by regulating Nrf2 signaling [115].

### 1.5. ROS and Ataxia

Ataxia is the cardinal symptom of genetic disorders or secondary lesions affecting the central nervous system. It is characterized by the progressive and inevitable loss of muscle control, associated with a difficulty in executing voluntary movements. There are many forms of ataxia, classified according to the affected anatomical region, the triggering cause, the age of onset, and other features. Friedrich’s Ataxia is probably the most common form of this type of neurological diseases. Friedreich ataxia is a genetic disease caused by the deficiency of frataxin, a mitochondrial protein supposed to work such as a metal chaperone and iron storage protein [116]. Frataxin-deficient cells were often observed to be highly sensitive to oxidizing agents [117,118]. Some authors have proposed that such sensitivity is associated to weakened biosynthesis of iron-sulfur centers. Thus, a vicious cycle would be generated in which the insufficient formation of iron-sulfur clusters would enhance mitochondrial free iron that, in turn, provokes ROS production through Fenton reaction. Thereafter, ROS would further impair iron-sulfur clusters, driving to the formation of more free iron. However, looking at the mitochondrial iron pools, no differences were observed between controls or Friedrick’s ataxia in human lymphoblasts or fibroblasts [119]. Nevertheless, some authors have suggested that oxidative stress could occur upstream (and not be the consequence) of iron-sulfur deficiency [116]. Undoubtedly, frataxin deficiency is reported to affect cell physiology at several levels, such as oxidative stress causing protein and DNA dysfunctions. Moreover, alterations in lipid metabolism have been observed in several models of the disease [117]. Therefore, increased oxidative stress together with chronic reduction of endogenous antioxidants causes neurodegeneration and affects cell survival. Indeed, it has been observed a significant increase in ROS and lipid peroxidation and a lower level of GSH in cerebellar granule neurons [120,121]. Moreover, Armstrong et al. highlighted that any alteration in the OXPHOS chain, caused by frataxin deficiency, could induce electron leakage and thus produce ROS [122]. 

All these effects due to an increased ROS production linked to mitochondrial dysfunction, as well as more significant oxidative damage have been shown likewise in other ataxia cell models [123,124,125].

## 2. Mesenchymal Stem Cells Potential in Neuronal Differentiation 

To clarify the choice to focus our attention on mesenchymal stem cells application in neurodegenerative diseases, a whole picture of different stem cells characteristics, with advantages and drawbacks, is reported below. Stem cells can be categorized as embryonic stem cells (ESCs), induced pluripotent stem cells (iPSCs), fetal stem cells (FSCs), perinatal stem cells (PSCs), and adult stem cells (ASCs) [126]. ESCs are pluripotent stem cells isolated from the inner cell mass of blastocysts before the stage of gastrulation [127,128]. They have advantages for self-renewal and differentiation even if the formation of in vivo teratomas is a problematic point. An additional complication is to find patient-matched ESCs. Moreover, there are many ethical concerns since the isolations of ESCs implies the destruction or manipulation of human embryo [129]. Interestingly, the more recent use of induced pluripotent stem cells (iPSCs) carries similar advantage and concerns, since iPSCs show comparable pluripotency potential to ESCs, but, being created from adult somatic cells through epigenetic reprogramming, ethical problems are avoided [130] and it also makes possible personalized cell therapy. However, generation of pluripotency and some of the procedures of reprogramming itself cause genomic instability affecting cell integrity. Moreover, the reprogramming factors (such as c-Myc) are known to be proto-oncogenes [131].

Fetal stem cells (FSCs) are obtained from fetal tissues of the beginning of the second trimester, such as amniotic fluid stem cells; therefore, they exhibit a lower stemness property than ESCs [132], even if they are defined as broadly multipotent [133]. Fetal as well as perinatal stem cells are of high interest since these cells can be easily isolated from discarded samples at the end of prenatal screening during second trimester or as clinical waste material after birth. Interestingly, FSCs share characteristics with both adult and embryonic stem cells, still maintaining high self-renewal, but without tumorigenic and ethical concerns [134].

Finally, adult stem cells (ASCs), from both allogeneic and autologous sources, are multipotent cells found in most adult tissues, even if in different amount, where they are responsible of tissue regeneration [135]. However, the age of the donor is linked to progressive decline of stem cell function [136].

Multipotent stem cells resident into the brain, neural stem cells (NSCs), are highly expandable ex vivo, but the transplantation of developing NSCs into the adult brains implies numerous ethical, scientific, and legislative hurdles [137]. Moreover, human neurogenesis drastically diminishes during aging and, as a consequence, NSC and neural precursor cells proliferation and differentiation are nearly undetectable [138] or they occur to a lesser extent and are restricted to defined areas, such as circumventricular organs [139] and the dentate gyrus [140].

With the purpose of stem cell therapy application for replacing degenerated cells in neurodegenerative disorders and NS lesions, mesenchymal stem cells (MSCs), adult stem cells generated from the mesoderm and neuroectoderm [141], show an elevated differentiation plasticity. These cells can be obtained from adult tissues, such as adipose tissue and bone marrow, but also from fetal tissues, such as umbilical cord blood, among other tissues. These stem cells have remarkable therapeutic properties and different advantages in respect to other adult stem cells such as NSCs. For example, not only fetal but also adult mesenchymal stem cells derived from adipose tissue exhibit triploblastic differentiation, thus pluripotency, both spontaneously and under media-specific induction, without forming teratomas [142]. Moreover, MSCs migrate towards inflammatory areas due to the expression of chemokine receptors [143], and, thanks to the immunomodulatory capacity, the use of MSCs might elude the side effects of the immunosuppressive treatment used with NSCs [144]. In addition, gestational tissues contain MSCs stem cells that inhibit or limit inflammatory responses and promote anti-inflammatory pathway. Perinatal stem cells express and secrete an arsenal of anti-inflammatory and pro-regenerative cytokines, involved in extracellular matrix remodeling and angiogenesis including macrophage inhibitory protein and hepatocyte growth factor [145]. Thus, MSCs isolated from fetal tissues may be considered a good alternative to adult MSCs for clinical applications, thanks to the previously reported advantages and because, unlike adult stem cells, they have the benefit of carrying fewer environmental induced mutations due to their early gestational derivation.

Nevertheless, when implanted in the injured region, beside differentiation and integration onto the damaged tissue, therapeutic effect of MSCs could be due to different mechanisms: secretion of neurotrophic factors [146], astroglial activation and neurogenesis induction [147], axon growth [148], enhancement of synaptic and cortical interhemispheric connections [149], immunomodulatory and anti-inflammatory properties [150], and decrease of apoptosis and oxidative stress [151]. Many cells types secrete exosomes as key players in cell–cell communication: exosomes contain many bioactive molecules and can regulate the expression of genes associated to neuronal development and transcription factors [152]. Exosomes can be considered as a tool to improve the modification of adult stem cells, namely endogenous NSCs, even eliminating the need for stem cell injection into the brain [153]. Thus, administration of conditioned media derived from MSCs could contribute in maintaining the increased paracrine factor gradient between the diseased organ and the stem cell niche in order to speed up the process of recovery. Based on the principle of paracrine signaling mechanism, the secretome of the MSCs is a rich source of the paracrine factors [154]. However, regarding the capability of MSCs to differentiate into neuronal cells, many studies have been conducted, by using several differentiation protocols, with contrasting results. Culture media with growth factors and other substances promote the differentiation of MSCs in vitro towards various neuronal cell types displaying differences in morphology and functions [155]. Indeed, various media have been used for neurogenic differentiation. Table 1 shows the supplements most frequently used and the neuronal cell types obtained after differentiation of MSCs.

Considering all these aspects, MSCs are simpler to use in the clinical practice, also compared to ESCs, by the easy preparation and by immunologic and ethic privileges. Moreover, MSCs and their secretome seem to be able to pass the blood–brain barrier, avoiding invasively intracerebral transplantation [156]. For these reasons, US FDA approved their use in clinical treatment for many disorders, including age-related diseases such as neurodegenerative disorders. Cell therapy has been evidenced to alleviate symptoms or even to reverse disease progress in neurological disorders where classical pharmacological treatments are no longer sufficient or are not available [157]. 

However, in their review, Peng et al. concluded that more data are needed to demonstrate the efficacy of the MSC-based cellular therapies in the treatment of neurodegenerative disorders [158]. Moreover, the studies reviewed by Peng et al. [158] already suggested that MSCs induced endogenous neuronal growth by a paracrine action, reduced free radicals levels, and regulated inflammation, thus stimulating functional recovery.

The observation that MSC secretome possesses antioxidant properties has also been proved in other contexts. In the injured liver, hypoxic conditioned media derived from adipose stem cells provided protection against ROS-related toxicity by increasing the expression of antioxidant enzymes, in part by releasing Nrf2 [159]. Bellei et al. [160] recently demonstrated that secreted molecules, obtained from adipose tissue, ameliorated the ability to dampen oxidative stress through a rise on intracellular antioxidant enzymes that improved cell proliferation in dermal and epidermal vitiligo cells in vitro. 

Based on these observations, in the following paragraphs, we report recent evidence on the role of MSCs or their secretome in counteracting neurodegenerative disorders by modulating oxidative stress. Table 2 and Table 3 summarize the in vitro and in vivo studies reported below.

## 3. Mesenchymal Stem Cells Contrast Neuronal Cell Damage Induced by Oxidative stress

Most of the literature suggests a beneficial effect of MSCs in neurodegeneration, demonstrating a molecular mechanism that involves the regulation of oxidative stress in vitro. For example, Baez-Jurado et al. [161] investigated the effect of conditioned medium (CM) of MSCs derived from human adipose tissue (AD) in a model of human astrocytes (T98G cells) subjected to scratch injury. Their findings demonstrated that CM-AD-MSC modulates mitochondrial dynamics and the respiratory chain, downregulates calcium in the cytoplasm, and settles the cytokines IL-2, IL-6, IL-8, IL-10, GM-CSF, and TNF-α. In parallel to these effects, a modulation of signaling pathways, such as AKT/pAKT and ERK1/2/pERK, occurs, and it can be linked to the intracellular localization of neuroglobin, mediating the protective effects of CM-AD-MSC through tuning of proteins implicated in survival pathways and oxidative stress. 

Mesenchymal stem cells, derived from the orbital adipose tissue, were demonstrated to possess neuroectodermal differentiation and repair ability in a recent study by Mawrie et al. [162]. In fact, also this particular type of MSCs abrogated neuronal cell damage induced by oxidative stress and expressed several neurotrophic factors, suggesting that the use of the MSCs and their secreted factors may represent a promising tool for neurological disease treatment.

The strategy to improve the antioxidant efficacy of MSC was explored in a recent paper by Wen et al. [163]. They investigated the antioxidant effects of secretions from BMSCs pretreated with berberin (BBR) on tert-butyl hydroperoxide (t-BHP)-damaged neurons, demonstrating that BBR can enhance BMSC viability and the secretion of nerve growth factor (NGF) and brain-derived neurotrophic factor (BDNF). These effects of conditioned medium (BBR-BMSC-CM) were related to a reduced ROS production, and may be due to an activation of the antioxidant proteins Keap1, Nrf2, and HO-1, and a decrease in the mitochondrial membrane potential. Thus, BBR-BMSC-CM ameliorated neuronal synaptic function and neuronal apoptosis by decreasing levels of the apoptotic proteins Bax/Bcl-2, cytochrome c, and cleaved caspase-3/caspase-3. 

To induce neurotoxicity, Palomares et al. [164] used an in vitro model of ec23/brain-derived neurotrophic factor (BDNF)-differentiated human SH-SY5Y neuron-like cells (SH-SY5Yd) exposed to H_2_O_2_. The effect of conditioned medium from human AD-MSCs was neuroprotective; indeed, stressed SH-SY5Y cells recovered normal axonal, electrophysiological features, and cell viability. The antioxidant capacity of AD-MSC conditioned medium and the presence of growth factors, namely, BDNF, glial cell line-derived neurotrophic factor, and transforming growth factor β1, are considered the involved therapeutic factors. However, the addition of the antioxidant agent N-acetyl cysteine to AD-MSC-CM eliminated its curative effect; this could be due to a strong reduction in ROS, in contrast to the moderate decrease in ROS produced by AD-MSC-CM alone. 

On the other hand, not all the studies suggest a positive effect of MSCs against neuron toxicity and ROS modulation. Indeed, when rat organotypic hippocampal cultures were exposed to CM obtained from MSCs isolated from rat bone marrow, cell toxicity in hippocampal cultures occurs. Furthermore, MSC-CM worsens cell death induced by glucose and oxygen deprivation. This negative effect was due to a marked glial cell activation after exposure of cultures to MSC-CM, as demonstrated by Horn et al. [165]. In fact, TNF-α and interleukin-6 levels were increased in the culture medium as well as ROS and iNOS, hinting that MSC-secreted factors induce reactive species generation and neuroinflammation in organotypic cultures of hippocampus. The involvement of microglial cells was also studied in a recent paper of Cao et al. [166] reaching the opposite conclusions. Specifically, the human bone marrow-derived mesenchymal stem cells (hBM-MSCs) significantly inhibited lipopolysaccharide (LPS) activation of microglial cells. In particular, these data indicate an inhibitory effect of hBM-MSCs on oxidative stress and pro-inflammatory mediators, involving the AMPK pathway in LPS-activated microglial cells. 

These results suggest that neuronal therapeutic effect of stem cells is due to not only the release of essential neurotrophic factors, but also to the maintenance of an appropriate redox state that preserves neuronal function. Moreover, appropriate cell culture conditions are necessary to maintain the properties of stem cells ex vivo for clinical applications taking into consideration the fundamental role of the cellular redox status [7,167].

In conclusion, a fine regulation of brain inflammation, associated to the rescue of essential cellular redox features in contrast to injury, makes CM-MSCs, more than MSCs, a promising protection tool for astrocytes and neurons in brain pathologies.

### 3.1. Mesenchymal Stem Cells Modulate Redox State in Alzheimer’s Disease

Neuronal cells treated with Aβ are widely used as an AD model as the extracellular deposition of Aβ protein plays a pivotal role in AD pathogenesis and progression. In a study by Lee et al. [168], cultured hippocampal neurons, treated with Aβ, were exposed to human umbilical cord blood-derived mesenchymal stem cells (hUCB-MSCs). hUCB-MSCs presence reduced apoptosis of hippocampal neurons induced by Aβ. Moreover, the authors showed that markers of oxidative stress, glial activation, and apoptosis levels declined in an acute AD mouse model. Interestingly, AD mice injected with hUCB-MSCs displayed cognitive rescue with improved learning/memory function. 

In a AβPP/PS1 mouse model of AD, intravenous infusion of hUCB-MSCs significantly improved spatial learning and alleviated memory decline, as demonstrated by Yang [169]. hUCB-MSC treatment increased glutathione (GSH) activity and ratio of GSH to oxidized glutathione as well as superoxide dismutase activity, while decreased malondialdehyde activity and protein carbonyl levels, suggesting that hUCB-MSC infusion alleviates oxidative stress in AβPP/PS1 mice. In addition, hUCB-MSC infusion reduced β-secretase 1 and CTFβ, thus reducing Aβ deposition in mice.

Furthermore, the secretome of MSCs, namely extracellular vesicles (EVs) derived from human Wharton’s jelly mesenchymal stem cells, is able to defend hippocampal neurons from oxidative stress and to avoid synapse damage prompted by amyloid-β oligomers. Indeed, Bodart-Santos et al. [170] recently demonstrated that hMSC-EVs were captured by hippocampal cells, and this uptake was improved in the presence of Aβ oligomers in the medium. Interestingly, neuroprotection by hMSC-EVs was related to the transfer of active antioxidant enzyme catalase contained in EVs.

The observation that MSC treatment has the potential to ameliorate Aβ pathology in AD model mice, modulating oxidative stress through regulation of microglial functions, was recently confirmed by Yokokawa et al. [171], both in vitro, by co-culturing the mouse microglial cell line MG6 with MSCs, and in vivo, using an AD mice (APdE9) model. MSCs induced the switch of microglial phenotype from M1 to M2 and reduced secretion of proinflammatory cytokines. Moreover, MSC transplantation enhanced microglia accumulation around Aβ deposits triggering microglial Aβ uptake and elimination. MSCs injected into the tail vein ameliorated spatial memory through a lower Aβ deposition in the cortex and hippocampus. To evaluate the redox state of the brain in vivo, they used electron paramagnetic resonance imaging, and they clearly demonstrated that MSC transplantation inhibited oxidative stress in this AD mice model. 

All these findings suggest that the regulation of redox state both inside neurons, inducing an increase of antioxidant enzyme level, and in the extracellular microenvironment, modulating the microglia activation, could prevent the progression of AD. Further studies on tau AD models are needed to confirm the redox state as a common key that is exploited by MSC to contrast this neurodegenerative disease.

### 3.2. Antioxidant Effects of Mesenchymal Stem Cells in Parkinson’s Disease

Mesenchymal stem cells and mouse embryonic stem cells are capable of generating neuronal precursors (NPs), which are a promising therapeutic strategy for neurological disorders due to their capacity to differentiate into functional astrocytes, oligodendrocytes, and mature neurons. Edwards Iii et al. [172] intravenously administered NPs in a 1-methyl-4-phenyl-1,2,3,6-tetrahydropyridine (MPTP) mouse model of PD. Previous research using intracerebral stem cell therapy for treatment of PD has provided promising results; however, this method is very invasive and is often associated with unacceptable side effects. These mice treated with NPs showed enhanced motor function and reduced neuroinflammatory response. It can be concluded that peripheral administration of stem cell-derived NPs could be used as valid therapy to treat PD brain pathology through the restoration of damaged motor function.

The mechanisms involved into the neuroprotective activity of MCSs were explored by using the conditioned media obtained from rat adipose tissue-derived MSCs (rAD-MSCs) in vitro models of PD neurodegeneration obtained exposing SH-SY5Y cells to pro-oxidizing agents such as hydrogen peroxide or the dopaminergic selective toxin 6-hydroxydopamine (6-OHDA) [173]. When neuroblastoma SH-SY5Y cells were exposed to CM of rAD-MSCs, cytotoxicity and ROS generation were reduced, through the upregulation of the antioxidant enzyme sirtuin 3 (SIRT3).

Notably, fetal stem cells, such as naïve human chorial villi (hCVCs) and cells derived from amniotic fluid (hAFCs), expressing some exclusive markers of neural stem/progenitor, such as nestin and connexin 43, also secrete BDNF and vascular endothelial growth factor. hCVC and hAFC populations are indeed composed of different cell lineages, including MSCs and cells with neural-like phenotypes. Calzarossa et al. [174] demonstrated that CMs derived from both cell types increased metabolic activity in a cell model of PD induced by 6-OHDA, but only hCVC-derived CM significantly diminished apoptosis induced by neurotoxin. The choice to use naïve cells instead of selecting stem cells population contained in chorial villi and amniotic fluid could be the reason for these contrasting results. However, they concluded that naïve hCVCs and hAFCs may contrast cell-neuronal damage by different mechanisms, and can be considered a promising stem cell therapy for neurodegenerative diseases including PD. 

Another study showed that conditioned medium, obtained from human menstrual blood-derived endometrial stem cells (MenSCs-CM), and not the cells itself, alleviated the symptoms of PD [175]. Human neuroblastoma SH-SY5Y cells were exposed to neurotoxicant 1-methyl-4-phenylpyridinium (MPP+) in order to obtain PD features. Then, pro-inflammation cytokines, oxidative stress, mitochondrial membrane potential (ΔΨm), cell viability, and cell apoptosis were assessed. MenSCs-CM demonstrated efficacy in contrasting MPP+ induced inflammation, ROS generation, ΔΨm loss, and decreased apoptosis and this protective effect could be related to various neuroprotective factors contained in MenSCs-CM.

In conclusion, the complexity of the stem cell secretome, including neuroprotective, neurotrophic, antiapoptotic, anti-inflammatory, and antioxidant molecules, can be considered the winning factors to counteract PD.

### 3.3. Mesenchymal Stem Cells Counteract Oxidative Stress in Other Brain Disorders

Damages to ganglion cells are often associated to other diseases, such as diabetes. Ezquer et al. [176] assessed that the intravitreal administration of adipose-derived MSCs avoided the loss of retinal ganglion cells in diabetic mice. Indeed, MSC injection significantly slowed down retinal ganglion cell loss. However, engrafted donor cells in the vitreous cavity did not differentiate into neural or perivascular-like cells, but their effect was to induce higher intraocular levels of several neurotrophic factors (basic fibroblast growth factor, nerve growth factor, and glial cell line-derived neurotrophic factor) and to lower the oxidative injury in the retina.

Similarly, Cui et al. in 2017 [177] explored whether bone mesenchymal stem cells (BMSCs) could prevent hydrogen peroxide-induced damage in retinal ganglion cells, by using immortalized retinal ganglion cells, RGC-5 cells. Exposure to BMSCs actively avoided retinal cell death, reducing H_2_O_2_-induced inflammatory factors IL-1β and TNF-α, downregulating intracellular oxidant factor malondialdehyde (MDA), and upregulating the intracellular antioxidant enzyme SOD and the expression of the neurotrophins CNTF and BDNF. Thus, BMSCs may induce defensive effect in RGC-5 cells against H_2_O_2_-induced injury enhancing the antioxidant potential, promoting neurotrophin expression and decreasing pro-inflammatory cytokine secretion.

It has been suggested that the method to obtain the best secretome for diabetic neuropathy (DN) treatment is by preconditioning MSCs that triggers an increase of the total antioxidant ability of the secretome. Oses et al. [178] demonstrated that the preconditioning of human AD-MSCs, with the iron chelator deferoxamine (DFX), increased the expression of the hypoxia inducible factor 1 alpha (HIF-1α), without effects on MSC morphology and survival. This was accompanied by an increased release of potent neuroprotective factors, such as nerve growth factor, glial cell-derived neurotrophic factor and neurotrophin-3, and cytokines with anti-inflammatory activity, such as IL-4 and IL-5. Moreover, DFX displayed neuroprotective effects also in an in vitro model of DN. Altogether, this study demonstrates that preconditioning with DFX improves therapeutic potential of AD-MSCs, suggesting this approach as a good strategy for DN treatment. As previously underlined, ALS is characterized by death of lower and upper motoneurons. Bonafede et al. [179] isolated exosomes from murine adipose-derived stromal cells and tested their efficacy on motoneuron-like NSC-34 cells expressing ALS mutations, as in vitro model of this disease. Exosomes demonstrated the ability to protect NSC-34 cells from oxidative damage, which is one of the central mechanisms of damage in ALS, and thus to increase cell viability. Maguire et al. [180] used the secretome obtained from four cell types, namely MSCs, fibroblast, neural stem cells, and astrocytes, demonstrating a slowdown in neuron and neural cell degeneration. In particular, sodium arsenite-induced oxidative stress in a human recombinant TDP-43 or FUS-tGFP U2OS cell line was used as in vitro model of ALS and frontotemporal dementia. This was significantly reduced by the exposure to the molecules released by MSCs. Furthermore, they also showed that the whole MSC secretome saves cortical neurons from glutamate toxicity, as demonstrated by better neurite outgrowth, reducing both LDH release and caspase 3/7 activity. On these bases, stem cells exosomes can also be a promising strategy in therapeutic applications for motoneuron diseases. 

Friedreich’s ataxia (FA) cells are particularly vulnerable to oxidative stress. Jones et al. [181] exposed FA-cells to oxidative stress in the presence of adipose stem cell-conditioned medium. Interestingly, AD-MSC-CM enhanced cell survival and upregulated oxidative-stress-associated genes such as frataxin, among other genes. Moreover, this work showed that neurotrophic factors, particularly BDNF, play a central role in enhancing cell survival contrasting oxidative stress.

Taking all together, the exposure to MSCs or their secretome can be considered as a promising therapeutic strategy to enhance antioxidant capacity and neurotrophin expression while inhibiting pro-inflammatory cytokine secretion, that are common aspects of neurodegenerative pathologies.

## 4. Conclusions

Neurodegenerative diseases represent a group of disorders characterized by a slow and chronic loss of different neuronal populations in the CNS. All of them possess a complex and multifactorial etiology and share common characteristic among which oxidative stress plays a fundamental role.

**Figure 4:**
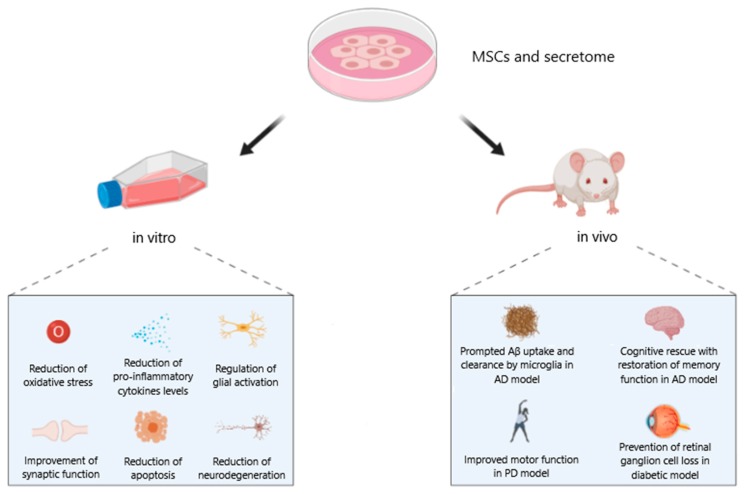
Schematic representation of the redox modulating demonstrated effects of mesenchymal stem cells in counteracting neurodegeneration in vitro and in vivo. Image created with BioRender.com.

In view of the complex multifactorial nature of neurodegenerative diseases, interventions concurrently targeting different risk factors and disease mechanisms at an early stage of the diseases are most likely to be successful. For this purpose, the contribution of stem cells has been widely explored over the past years and neuro-restoration seems to be a promising strategy (Figure 4). 

In particular the exposure to mesenchymal stem cells can be considered as a promising therapeutic tool for neurodegenerative diseases since MSCs:

- enhance antioxidant capacity;
- increase neurotrophin expression;
- inhibit pro-inflammatory cytokine secretion; and
- counteract microglial ROS production.

The main criticism is that the clinical application of stem cell therapy in counteracting neurodegeneration in humans is still questionable, generating both interests and alarms in the scientific community. The most interesting approach to overcome these criticisms could be to avoid cells injection employing only the secretome, namely extracellular vesicles, obtained from MSCs cultured in vitro and properly preconditioned in order to reach the best pool of bioactive molecules. In this sense, since EVs are apparently well-tolerated, their use paves the way for innovative and more efficient therapies based in nanomedicine, avoiding the putative side effects associated with stem cell transplantation. However, differences in MSCs and secretome can occur, depending on the tissue source, and characteristics of the donor, such as age.

Therefore, further studies are needed to identify a tailored approach for each neurodegenerative disease in order to design more effective therapeutic stem cell strategies to prevent a broad range of neurodegenerative disorders. Indeed, in the future, it will be necessary to challenge several, and still unsolved, problems such as: (1) the appropriate number of stem cells or exosomes to transplant; (2) the optimal timing of transplantation; (3) the correct disease stage for cell transplantation; and (4) the evaluation of the therapeutic effectiveness of the different kind of MSCs in terms of safety, function recovery, and motor and cognitive improvement.

## Figures and Tables

**Table 1:** Neurogenic differentiation protocols.

Compounds	Neuronal Markers	Types of Neuronal Cells	Types of MSC	Ref.
10% AP-g-CH/GL/AG scaffold + RA, Shh, BDNF, GDNF	Hb-9 Islet-1 ChAT TCP	Motor neuron-like cells	hOE-MSCs	[182]
Shh, bFGF, FGF8 and BDNF in free-serum condition	TUJ1 (28–60% of cells) TH NSE EN1 GLI1 NURR1 VMAT2 GIRK2	Dopaminergic neurons	hADSCs	[183]
PVA/SA nanofibers + beta carotene	Morphology MAP-2	Neural-like cells	hBMSCs	[184]
PEMF	Morphology (elongated spindle shape) CD104 S100 GFAP laminin P75NTR	Schwann-like cells	hDPSCs	[185]
Lamin and PDGF-BB	Nestin MAP2 β-tubulin III Nerofilament-M	Neuron-like cells	hUSCs	[186]
Phase I: trans-RA Phase II: N2B27	GAPDH Nestin Sox1 β-tubulin III NF200	Phase I: NPCs Phase II: mature cells of the neural lineage	MESC lines: A2lox and 129	[187]
Serum-free + BDNF + NT-3/neurotrophins	β-tubulin III Nestin Connexin (Cx26, Cx43) Pannexin (Pnx1) NTRK2, NTRK3 NGF, BDNF, NT-3 expression	Central NSC and peripheral neural crest stem cells	hDPSCs	[188]
10% FBS, Retinoic acid	GFAP, β-III tubulin, CNPase, MAP2, NeuN, synapsines, S100, PMP22	Neuron-like cells, oligodendrocyte-like cells	hAFSC	[189]
Heparin mix with FGF	ChAT genes expression β-tubulin III MAP2 Sox1	Dopaminergic neurons	hADSCs	[190]
Retinoic acid mix with EGF, FGF2, BMP-9	TH genes expression β-tubulin III MAP2	Cholinergic neurons	hADSCs	[190]
BME + NGF	NF-68, NF-160, NF-200 NeuroD ChAT MAP-2 synapsin-1	Choliergic neurons	mBMSCs	[191]
Melatonine	β-tubulin III TH NURR1 GFAP_decrease_ CD29, CD45, CD73, CD90, CD105_decrease_ DAT	Dopaminergic neurons	hAF-MSCs	[192]
SHH + RA	Neuron-like morphology NF-M TuJ-1 vimentin synapsin membrane potential (−70/−65 mV)	Cholinergic neurons	Monkey BMSCs	[193]
Cyclic ketamine	Filamin-B Rac1 thioredoxine peroxiredoxin-1 Gelsolin Galectin-3	Neurons	hADSC	[194]
BDNF + FGF2	MAP2 TH NGN2 PITX3 DAT synaptophysin Kv4.2 SCN5A	Dopaminergic neurons	hDPSCs	[195]
TMP	Neuronal morphology MAP-2 NSE Ngn1 NeuroD Mash1	Neurons	mBMSCs	[196]
e-CSF	β-tubulin III neurite outgrowth assay	Neuronal-like cells	m-ADSCs	[197]

AP-g-CH/GL/AG scaffold, chitosan-graft-aniline pentamer, gelatin, agarose; hOE-MSCs, human olfactory ecto-mesenchymal stem cells; hADSC, human adipose tissue-derived stem cells; NSE, neuron specific enolase; EN1, engriled homebox 1; GLI1, GLI family zinc finger 1; NURR1, nuclear receptor-related factor 1; VMAT2, vesicular monoaminetransporter 2; GIRK2, G-protein-regulated inward-rectifier potassium channel 2; PVA/SA, polyvinyl alcohol/sulphated alginate; hBMSCs, human bone marrow stem cells; PEMF, used electromagnetic field; hDPSCs, human dental pulp stem cells; PDGF-BB, platelet-derived growth factor BB; hUSCs, human urine-derived stem cells; RA, retinoic acid; mESC, mouse embryonic stem cell; NPCs, neural precursor cells; mBMSCs, murine bone marrow stem cells; BME, β-mercaptoethanol; NGF, nerve growth factor; ChAT, choline acetyl transferase; DAT, dopamine transporter; TMP, tetramethylpyrazine; MAP-2, microtubule-associated protein 2; NSE, neuron-specific enolase; Ngn1, neurogenin 1; NeuroD, neuronal differentiation 1; Mash1, mammalian achaete-scute homolog 1; e-CSF, embryonic cerebrospinal fluid.

**Table 2:** MSC studies on in vitro models of neurodegenerative diseases.

In Vitro Model	Analyzed Pathways	MSC Sources	Redox Pathway Involved	Ref.
Primary hippocampal cultures exposed to Aβ	TUNEL staining DHE staining	hUCB-MSCs co-culture	Reduction of oxidative stress and apoptosis level	[168]
(LPS)-activated microglial cells	AMPK pathway TNF-α, IL-1β, IL-6, PEG2, NADPH oxidase activity, ROS, MDA, SOD, GSH-Px	hBM-MSCs co-culture	Inhibitory effect on the pro-inflammatory mediators and oxidative stress by AMPK pathway	[166]
Neuroectodermal lineage cells treated with H_2_O_2_	NeuroD and Nestin expression, Bax, Bcl-2	OAMSCs-CM containing neurotrophic factors	Abrogation of neuronal cell damage induced by oxidative stress	[162]
RGC-5 cells (diabetic retinopathy (DR))	Cell viability IL-1β and TNF-α MDA and antioxidant enzymes (such as SOD) BDNF CNTF	mBMSCs co-culture	Reduction of H_2_O_2_-induced inflammatory factor, downregulation of intracellular oxidant factor (MDA) and upregulation of intracellular antioxidant factor (SOD), increase of neurotrophins expression	[177]
6-OHDA-induced PD cell model	Cell viability	hCVC-CM (nestin, connexin 43, VEGF)	Reduction of neurotoxin-induced apoptosis, enhancement of cell-recovery following neuronal damage	[174]
NSC-34 cells expressing ALS mutation (in vitro model of ALS)	H_2_O_2_-induced cytotoxicity	mADSC-exo	Neuroprotective effect of low concentration of exosomes on cell viability and protection from oxidative damage	[179]
FA cells model (periodontal ligament cells from FA patient)	CD44, CD34, CD-90 cleaved caspase-3 Oxidative-stress-related genes (such as frataxin) BDNF	hADSC-CM	Change of transcription levels of oxidative-stress-related genes avoiding cellular degeneration	[181]
SH-SY5Y (MPP+ for inducing PD)	Cell viability pro-inflammatory cytokines, mitochondrial membrane potential (Δψm) oxidative stress (DHE assay) cell apoptosis	hMenSC-CM	Increase of cell viability, reduction of MPP+ induced inflammation, Δψm loss, ROS generation and cells numbers in late apoptosis stage	[175]
SH-SY5Y (PD)	ROS detection (DCFDA) SOD2 Hsp70 SIRT1 SIRT3	RAA-MSC-CM	Reduction of mortality and ROS generation Upregulation of the antioxidant enzyme SIRT3 (SIRT1 and Hsp70 unchanged)	[173]
SH-SY5Yd exposed to H_2_O_2_	Cell viability assay ROS detection (DCFDA) Morphological analysis	ASC-CM	Moderate reduction of ROS and maintenance of an appropriate redox state to preserve neuronal function	[164]
Rat organotypic hippocampal cultures	GFAP Microglial cells detection (isolectin-B_4_) TNF-α, IL-6 ROS measurement (DCF-DA) iNOS	rBMSC-CM	Induction of glial activation Increase of ROS generation and neuroinflammation	[165]
Primary rat hippocampal cultures exposed to AβOs	ROS measurement (DCF-DA) Catalase detection	hMSC-EVs	Protection from oxidative stress and synapse damage induced by AβOs Reduction (via catalase activity) of abnormal ROS production and propagation of astrocyte neurotoxic effects induced by AβOs	[170]
Human recombinant TDP-43-tGFP U2OS cell line exposed to sodium arsenite	FUS TDP-43 Cell viability caspase-3 LDH release	hADSC-CM	Inhibition of stress granule number Rescue from glutamate toxicity, reduction of LDH release and caspase 3/7 activity	[180]
T98G cells (human astrocytes) subjected to scratch injury	IL-2, IL-6, IL-8, IL-10, TNF-α ROS determination (DHE) membrane potential (Δψm) Ca^2+^ intracellular GM-CSF AKT/pAKT ERK1/ERK2/pERK Ngb localization	hMSAC-CM	Modulation of localization and expression of neuroglobin Protective effects through regulation of proteins involved in survival pathways and oxidative stress	[161]
Rat DGR neurons	HIF-1α, NGF, neuroptophin-3, glial cell-derived neurotrophic factor, IL-4, IL-5, VEGF, angiopoietin 1	hAD-MSC secretome	DFX (iron chelator deferoxamine) preconditioning significantly increased the total antioxidant capacity of the MSC secretome	[178]

hUCB-MSCs, human umbilical cord blood-derived mesenchymal stem cells; hBM-MSCs, human bone marrow-derived mesenchymal stem cells; hOAMSCs, human orbital adipose tissue-derived mesenchymal stem cells; mBMSCs, murine bone marrow stem cells; 6-OHDA, 6-oxidopamine; hCVC, human chorionic villi cells; ALS, amyotrophic lateral sclerosis; mADSC, murine adipose-derived stem cell; FA, Friedreich’s ataxia; hADSC, human adipose-derived stem cell; MPP, 1-methyl-4-phenylpyridinium; DCFDA, 2′-7′dichlorofluorescin diacetate; hMenSC, human menstrual blood-derived endometrial stem cell; RAA-MSC, rat adipose tissue-derived MSC; rBMSC, rat bone marrow stem cells.

**Table 3:** MSC studies on in viivo models of neurodegenerative diseases.

In Vivo Model	Analyzed Pathways	MSC Sources	Redox Pathway Involved	Ref.
Diabetic retinopathy mouse model	NGF bFGF GDNF	AD-MSC injection (robust expression of SOD1, SOD2, CAT, GPX1)	Reduction of oxidative damage: small reduction in the ROS level but strong reduction in lipid peroxidation levels	[176]
PD mouse model (C57BL/6J MPTP-PD induced model)	Behavioral assessment (Wire Hang and Rotarod) GFAP and BLBP-expression Iba-1	NPs injection obtained from ESCs and MSCs	Amelioration of the motor symptoms Significantly reduction of reactive astroglia and microglia cells Reduction of astroglia and macrophages	[172]
AD model mice (APdE9)	MWM test Aβ deposition Iba-1 CD14-positive microglia Aβ_1-40_ and Aβ_1-42_ ELISA assay EPR imaging system	MSC injection (tail vein)	Amelioration of oxidative stress through upregulation of microglia expression Promotion of microglial Aβ uptake and clearance Decrease of Aβ-dependent ROS production, thereby improving the redox balance	[171]
AβPP/PS1 mouse model of AD	Reduction of β-secretase 1 and CTFβ, and Aβ deposition	hUCB-MSCs vain infusion	Increased GSH and SOD activity. Decreased malondialdehyde activity and protein carbonyl level	[169]
C57BL/6J mice (acute Aβ-induced AD model)	MWM test DHE staining TUNEL staining GFAP staining Iba-1 staining	hUCB-MSCs transplant	Recover of impaired memory function Inhibition of oxidative stress and of glial activation that mediates apoptosis	[168]

hADSC, human adipose-derived stem cell; NPs, neuronal precursors; ESCs, embryonic stem cells; MSCs, mesenchymal stem cells; hUCB-MSCs, human umbilical cord blood-derived mesenchymal stem cells; GSH, glutathione; SOD, superoxide dismutase.

## Abbreviations

6-OHDA	6-hydroxydopamine
AD	Alzheimer’s disease
ADSC	adipose-derived stem cell
AGEs	advanced glycation end products
ALS	amyotrophic lateral sclerosis
APP	amyloid-protein precursor
ARE	antioxidant response element
ASCs	adult stem cells
BDNF	brain-derived neurotrophic factor
BME	β-mercaptoethanol
BM-MSCs	bone marrow-derived mesenchymal stem cells
CM	conditioned medium
CNS	central nervous system
CVC	chorionic villi cells
DA	dopamine
DAT	DA transporter
DCFH-DA	2′-7′dichlorofluorescin diacetate
DPSC	dental pulp stem cells
e-CSF	embryonic cerebrospinal fluid
ESCs	embryonic stem cells
FA	Friedreich’s ataxia
FSCs	fetal stem cells
GSH	glutathione
HD	Huntington’s disease
HIF-1α	hypoxia inducible factor 1 alpha
iPSCs	induced pluripotent stem cells
MAP-2	microtubule-associated protein 2
MDA	malondialdehyde
MenSC	menstrual blood-derived endometrial stem cell
MSCs	mesenchymal stem cells
NeuroD	neuronal differentiation 1
NFTs	neurofibrillary tangles
NGF	nerve growth factor
Ngn1	neurogenin 1
NO	nitric oxide
NOS	nitric oxide synthase
NOXs	NADPH oxidases
NPCs	neural precursor cells
NPs	neuronal precursors
Nrf2	nuclear factor erythroid 2-related factor 2
